# A c-Fos activation map in nitroglycerin/levcromakalim-induced models of migraine

**DOI:** 10.1186/s10194-022-01496-8

**Published:** 2022-09-30

**Authors:** Shouyi Wu, Xiao Ren, Chenlu Zhu, Wei Wang, Kaibo Zhang, Zhilei Li, Xuejiao Liu, Yonggang Wang

**Affiliations:** 1 Department of Neurology, Lanzhou University Second Hospital, Cuiying Gate, No. 82Linxia Road, Chengguan District, Lanzhou, 730000 China; 2 Department of Neurology, The First Affiliated Hospital of Nanchang University, 17 Yongwaizheng Street, Nanchang, 330006 China; 3 Headache Center, Department of Neurology, Beijing Tiantan Hospital, Capital Medical University, No.119 South Fourth Ring West Road, FengtaiDistrict, Beijing, 100070 China

**Keywords:** Chronic migraine, Migraine-like pain, CGRP, c-Fos

## Abstract

**Background:**

Chronic migraine is a common and highly disabling disorder. Functional MRI has indicated that abnormal brain region activation is linked with chronic migraine. Drugs targeting the calcitonin gene-related peptide (CGRP) or its receptor have been reported to be efficient for treating chronic migraine. The CGRP signaling was also shared in two types of chronic migraine models (CMMs). However, it remains unclear whether the activation of specific brain regions could contribute to persistent behavioral sensitization, and CGRP receptor antagonists relieve migraine-like pain in CMMs by altering specific brain region activation. Therefore, it’s of great interest to investigate brain activation pattern and the effect of olcegepant (a CGRP receptor-specific antagonist) treatment on alleviating hyperalgesia by altering brain activation in two CMMs, and provide a reference for future research on neural circuits.

**Methods:**

Repeated administration of nitroglycerin (NTG) or levcromakalim (LEV) was conducted to stimulate human migraine-like pain and establish two types of CMMs in mice. Mechanical hypersensitivity was evaluated by using the von Frey filament test. Then, we evaluated the activation of different brain regions with c-Fos and NeuN staining. Olcegepant was administered to explore its effect on mechanical hyperalgesia and brain region activation.

**Results:**

In two CMMs, acute and basal mechanical hyperalgesia was observed, and olcegepant alleviated mechanical hyperalgesia. In the NTG-induced CMM, the medial prefrontal cortex (mPFC), anterior cingulate cortex (ACC), and the caudal part of the spinal trigeminal nucleus (Sp5c) showed a significant increase of c-Fos expression in the NTG group (*p* < 0.05), while pre-treatment with olcegepant reduced c-Fos expression compared with NTG group (*p* < 0.05). No significant difference of c-Fos expression was found in the paraventricular thalamic nucleus (PVT) and ventrolateral periaqueductal gray (vlPAG) between the vehicle control and NTG group (*p* > 0.05). In the LEV-induced CMM, mPFC, PVT, and Sp5c showed a significant increase of c-Fos expression between vehicle control and LEV group, and olcegepant reduced c-Fos expression (*p* < 0.05). No significant difference in c-Fos expression was found in vlPAG and ACC (*p* > 0.05).

**Conclusions:**

Our study demonstrated the activation of mPFC and Sp5c in two CMMs. Olcegepant may alleviate hyperalgesia of the hind paw and periorbital area by attenuating brain activation in CMMs.

**Supplementary Information:**

The online version contains supplementary material available at 10.1186/s10194-022-01496-8.

## Background

Migraine is one of the most common types of primary headache disorders and represents a brain state of altered excitability [1–3]. Current evidence suggests that about 3% of patients with episodic migraine annually progress to chronic migraine (CM) [4]. Recent neuroimaging studies showed structural and functional changes in the cortex [5–12], basal ganglia [7–10], thalamus [6, 12], hypothalamus [13, 14], and brainstem [14, 15] in patients with chronic migraine. Thus, it’s of great interest to explore how neurons are activated in these brain regions. However, in chronic migraine models (CMMs), including the repeated dural application of inflammatory soup, and chronic systemic infusion of nitroglycerin (NTG) [16, 17], studies investigating brain activation remain scarce [18].

In experimental models of migraine in humans, migraine could be triggered by various compounds such as nitric oxide (NO) donor NTG, calcitonin gene-related peptide (CGRP), phosphodiesterase 3 (PDE3) inhibitor cilostazol, and ATP-sensitive potassium (K_ATP_) channel opener levcromakalim (LEV) [19–21]. NTG causes increased intracellular cGMP and CGRP causes increased intracellular cAMP. Activation of cAMP and cGMP-mediated pathways results in the opening of K_ATP_ channels, so the modulation of nociceptive transmission by K_ATP_ channel may be a final common pathway in the genesis of a migraine attack [22]. Meanwhile, repeated systemic administration of LEV also induced hind paw and periorbital hyperalgesia in a mouse model, and CGRP signaling was shared in NTG and LEV-induced CMMs [23, 24]. However, whether the activation of specific brain regions could contribute to persistent behavioral sensitization remains unclear, and whether similar activation of brain regions occurs in two CMMs is still unknown. Moreover, it has been reported that gepants (CGRP receptor antagonists) are effective for the acute and preventative treatment of migraine [25]. In CMMs, olcegepant (OLC), a selective CGRP receptor antagonist, significantly alleviates mechanical hypersensitivity [23, 24, 26]. However, it is still unknown whether OLC can alter brain activation to relieve migraine-like pain. To address this question, two CMMs were established to investigate the activation of brain regions by NTG or LEV treatment and the effect of OLC treatment on brain activation by quantifying the expression levels of c-Fos, an immediate-early gene (IEG) widely used for brain activity mapping [27].

## Materials and methods

### Animals

All experiments with animals were approved by the Animal Ethics Committee in Lanzhou University Second Hospital and carried out according to the criteria outlined in the “Guide for the Care and Use of Laboratory Animals” prepared by the National Academy of Sciences and published by the National Institutes of Health. The sample size of this study was determined based on previous studies [28, 29]. In this experiment, adult male C57BL/6 J mice (18-26 g) were purchased from the Experimental Animal Center of Lanzhou Veterinary Research Institute. All animals were housed under standard conditions with a 12 h light/dark cycle, controlled room temperature, and standard rodent chow diet. In total, 48 male mice were used in the study. Mice were randomly assigned to different experimental groups with 8 male mice. Before all experiments started, mice were given one week to adapt to the experimental environment.

### Chronic migraine models

For the establishment of NTG-induced CMM, a stock solution of 5 mg/ml NTG (Beijing Yimin, China), containing 30% propylene glycol and 30% alcohol, was dissolved in water. Prior to the injection, NTG was freshly diluted to 1 mg/ml with 0.9% saline and administered intraperitoneally (i.p.) at a dose of 10 mg/kg every other day for 9 days, based on the literature [28, 29].

For the establishment of LEV-induced CMM, LEV (MedChemExpress, HY-14255), a K_ATP_ channel opener, was dissolved in dimethyl-sulfoxide (DMSO) to a final concentration of 5 mg/ml. Prior to the injection, LEV was freshly diluted to 0.1 mg/ml with 0.9% saline by ultrasonic instrument, administered intraperitoneally (i.p.) at a dose of 1 mg/kg every other day for 9 days, as previously described [23, 24].

### Drug administration

Olcegepant (MedChemExpress, HY-10095) was dissolved in DMSO to a final concentration of 5 mg/ml. Prior to the injection, OLC was freshly diluted to a final concentration of 0.1 mg/ml with 0.9% saline by ultrasonic instrument. OLC (1 mg/kg) was administered intraperitoneally (i.p.) 15 min prior to the LEV or NTG injection [23, 24]. Accordingly, 2% DMSO + 0.9% saline in the NTG-induced migraine model and 2% DMSO + 2% DMSO in the LEV-induced migraine model were used as the vehicle (VEH) control.

### Behavioral tests

All behavioral tests were conducted under low-light conditions between 9:00 and 15:00. Mice were habituated to the behavioral testing room for 2 days prior to behavioral tests. The experiment was double-blinded designed and all data were analyzed by another blinded observer. Previous studies showed that LEV/NTG-induced hyperalgesia was most pronounced 2 h after injection [24, 34]. Thus, the post-treatment mechanical threshold was measured 2 h after NTG or LEV injection (acute hyperalgesia) on each injection day. The basal mechanical threshold (basal hyperalgesia) was measured prior to the VEH, NTG, or LEV injection. For the periorbital mechanical threshold test, the mouse was put into a 4 oz. cup and allowed to adapt for 15 min [28, 29]. Von Frey monofilaments (range from 0.008 to 2 g) were applied perpendicularly to the periorbital region with the up-down method to assess the mechanical threshold. A positive response in the periorbital test was defined as quick retraction of the head from the stimulation or scratching the face with the ipsilateral forepaw. The periorbital test was performed every 4 days to avoid sensitizing the mice [23]. Before the hind paw mechanical threshold test, mice were placed on wire grid floors in clear plexiglass chambers (L X W X H: 10 X 7 X 7 cm) and allowed to habituate for 30 min [23, 24]. Von Frey monofilaments was applied as described above. A positive response in the hind paw test was defined as withdrawal, shaking, or licking of the paw [30, 31]. Finally, a 50% mechanical pain threshold was calculated by the online tool at https://bioapps.shinyapps.io/von-Frey-app/ [32].

### Immunofluorescence staining

After the last behavioral test on day 9, in order to reduce bias caused by a small sample size, we randomly selected 5 mice from 8 mice for immunostaining analysis in each group. Mice were deeply anesthetized with 1% pentobarbital and transcardially perfused with 1X phosphate-buffered saline (1 X PBS, pH = 7.4)), followed by 4% paraformaldehyde (PFA). Mouse brains were immediately dissected and postfixed overnight with 4% PFA at 4 °C. Then, the brain tissue was dehydrated in 20% and 30% sucrose solutions sequentially until the tissue sank. Tissue blocks were prepared by embedding them in Tissue-Tek O.C.T. Compound (Sakura 4583). The brain tissue was frozen and sectioned on a cryostat microtome (Leica, CM1950) to obtain 40 μm-thick sections. For immunostaining, the tissue sections were washed with 1 X PBS for a 5- minutes incubation and permeabilized with 0.4% Triton X-100 for 30 min at room temperature. After a wash with clean 1 X PBS buffer for a second 5 min incubation, the tissue sections were followed by incubation with rabbit anti-c-Fos antibody (1:500, Cell Signaling Technology) and mouse anti-NeuN (1:400, Abcam) in antibody dilution buffer (1% BSA, 1 X PBS, 0.4% Tryton X-100) overnight at 4 °C. After primary antibody incubation, the sections were washed for 2 times with 1 X PBS and incubated with following secondary antibodies for 2 h at room temperature: Goat anti-rabbit Alexa Fluor 488 and Goat anti-mouse Alexa Fluor 594 (1:400, Abcam). Cell nuclei were stained with 4′,6-diamidino-2-phenylindole (DAPI) at room temperature for 10 min. The sections were washed 2 times with 1 X PBS and coverslipped with 50% glycerol for imaging. Images were acquired with a confocal microscope under a 10 X objective (Leica SP8, Germany).

To quantify c-Fos^+^ and NeuN^+^ cells, in the medial prefrontal cortex (mPFC), anterior cingulate cortex (ACC), paraventricular thalamic nucleus (PVT), ventrolateral periaqueductal gray (vlPAG), and the caudal part of the spinal trigeminal nucleus (Sp5c) we manually outlined following the atlas of Paxinos (2nd edition from the mouse brain in stereotaxic coordinates) by ImageJ. Every 4 brain slices were selected for the density of c-Fos^+^ cells (c-Fos^+^/mm^2^) and the percentage of c-Fos^+^ cells in NeuN cells (c-Fos^+^ NeuN^+^/NeuN^+^ cell) in ImageJ. Due to the different depths of specific brain regions, thus the number of sections per brain region was different. In each mouse, 2 brain slices were quantified for mPFC, and 6 brain slices were quantified for PVT, vlPAG, and Sp5c, respectively.

### Statistical analysis

All data were presented as mean ± SEM. Data analysis was performed by PRISM 9.3 software (GraphPad, San Diego, CA). For behavioral test data, including drug administration and different time points, two-way ANOVA with the Tukey post hoc test was used. For c-Fos density quantifications data comparisons in three groups, one-way ANOVA followed by the Tukey post hoc test was used to determine statically significance. A *p*-value < 0.05 was defined as statistical significance.

## Results

### Olcegepant alleviated acute hyperalgesia but not basal hyperalgesia in the NTG-induced chronic migraine model

**Fig. 1:**
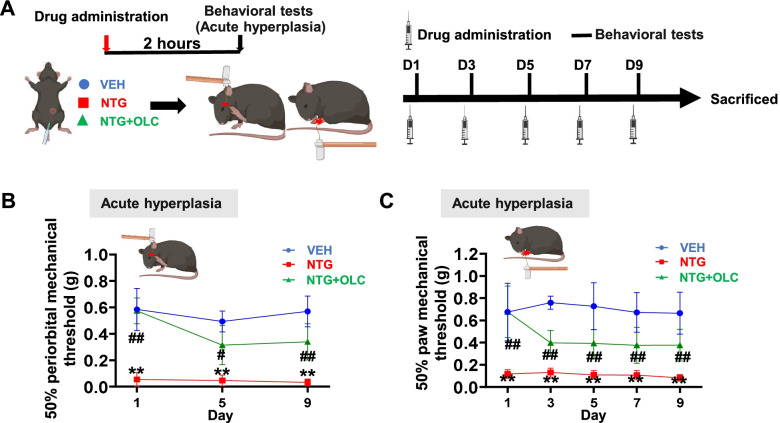
CGRP receptor antagonist (olcegepant) alleviated acute hyperalgesia in the NTG-induced chronic migraine model. **A.** Representative schematic diagrams and procedures for the behavioral tests. (Created with BioRender.com) **B-C.** Repeated NTG administration induced mechanical hyperalgesia of periorbital area (**B**) and hindpaw (**C**), alleviated by OLC. Two-way ANOVA with the Tukey post hoc tests; * *P* < 0.05, **P < 0.01, NTG group compared with the VEH group, *n* = 8/group; #*P* < 0.05, ## *P* < 0.01, NTG group compared with the NTG+OLC group, *n* = 8/group. Abbreviations: VEH, vehicle; NTG, nitroglycerin; OLC, olcegepant

Mice were injected with NTG every other day for 9 days to establish a CMM, and OLC was given 15 min before NTG injection in other treatment groups (Fig. 1A; Fig. S1A). The mechanical thresholds of the hind paw and periorbital area were significantly decreased in the NTG group compared with the VEH group. Interestingly, pre-treatment with OLC in the other NTG group alleviated acute hyperalgesia, but not basal hyperalgesia (*n* = 8/group, *p* < 0.01; Fig. 1B, C; Fig. S1B, C). These data indicated the successful establishment of the NTG-induced CMM in mice and OLC could alleviate acute hyperalgesia.

### c-Fos activation map of cortical structures, thalamic and brain stem structures in the NTG-induced chronic migraine model

**Fig. 2:**
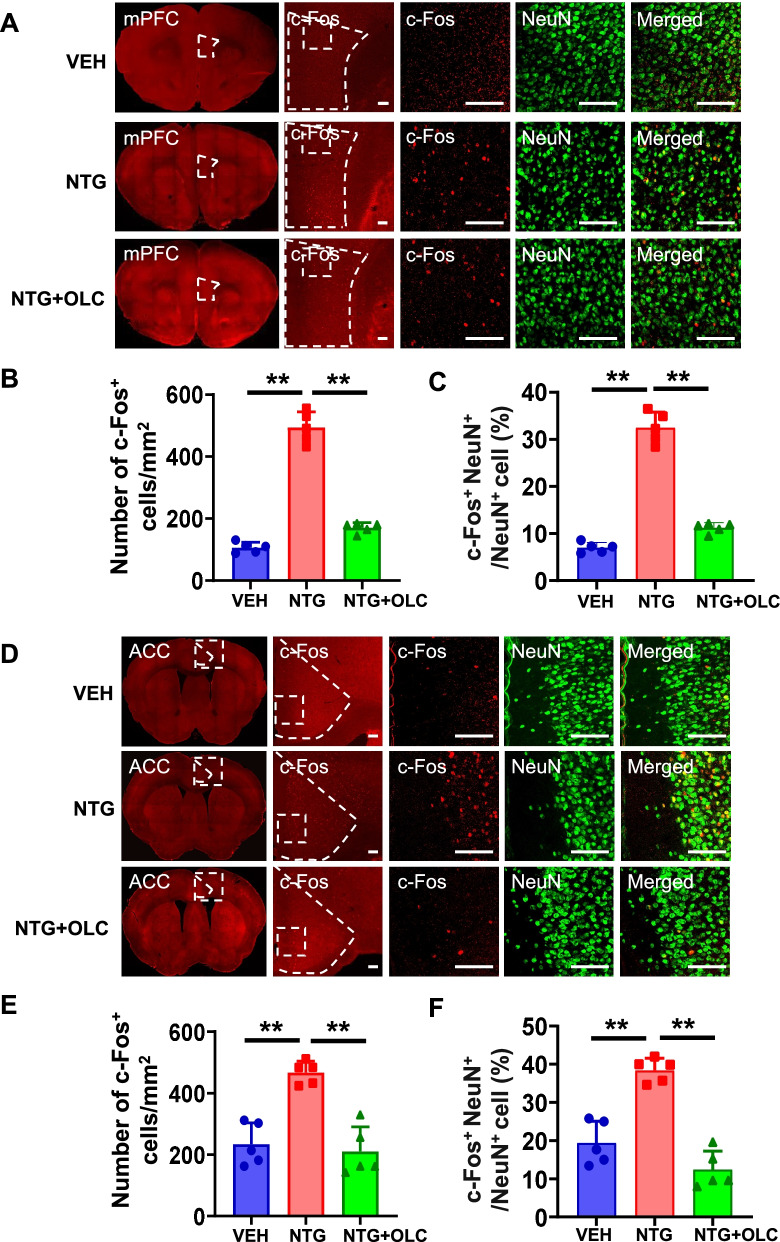
c-Fos activation map of cortical structures in the NTG-induced chronic migraine model. **A.** Representative pictures of c-Fos (red) and NeuN (green) immunofluorescence labeling in the mPFC. Scale bars =100 μm. **B-C.** mPFC showed a significant increase of c-Fos^+^ cells density (**B**) and the percentage of c-Fos + cells in NeuN ^+^ cells (**C**), alleviated by OLC, *n* = 5/group. **D.** Representative pictures of c-Fos (red) and NeuN (green) immunofluorescence labeling in the ACC. Scale bars =100 μm. **E–F.** ACC showed a significant increase of c-Fos^+^ cells density (**E**) and the percentage of c-Fos^+^ cells in NeuN cells (**F**), alleviated by OLC, *n* = 5/group. One-way ANOVA with the Tukey post hoc tests, * *P* < 0.05, **P < 0.01. Abbreviations: mPFC, medial prefrontal cortex; ACC, anterior cingulate cortex; OLC, olcegepant; NTG, nitroglycerin

**Fig. 3:**
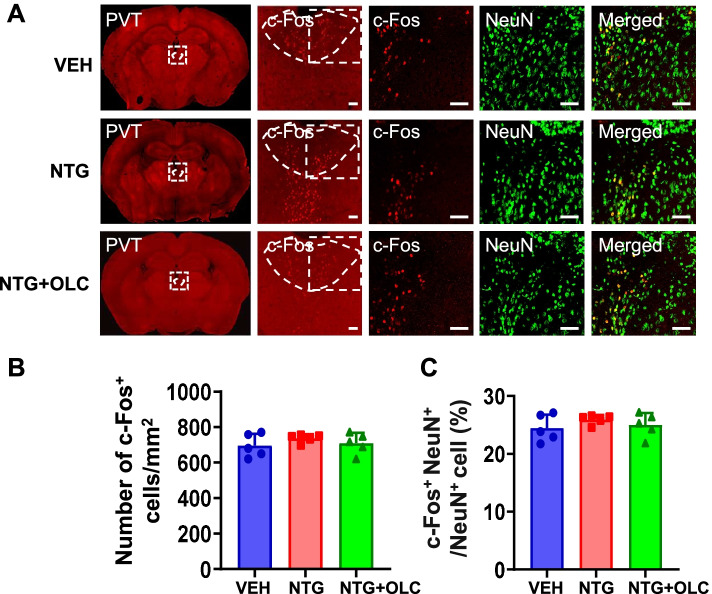
c-Fos activation map of thalamic structure in the NTG-induced chronic migraine model. **A**. Representative pictures of c-Fos (red) and NeuN (green) immunofluorescence labeling in the PVT. Scale bars =50 μm. **B-C**. In PVT, no significant differences in c-Fos^+^ cell density (**B**) and the percentage of c-Fos^+^ cells in NeuN^+^ cells (**C**) were found. *n* = 5/group. One-way ANOVA with the Tukey post hoc tests, * *P* < 0.05, **P < 0.01. Abbreviations: PVT, paraventricular thalamic nucleus. NTG, nitroglycerin

**Fig. 4:**
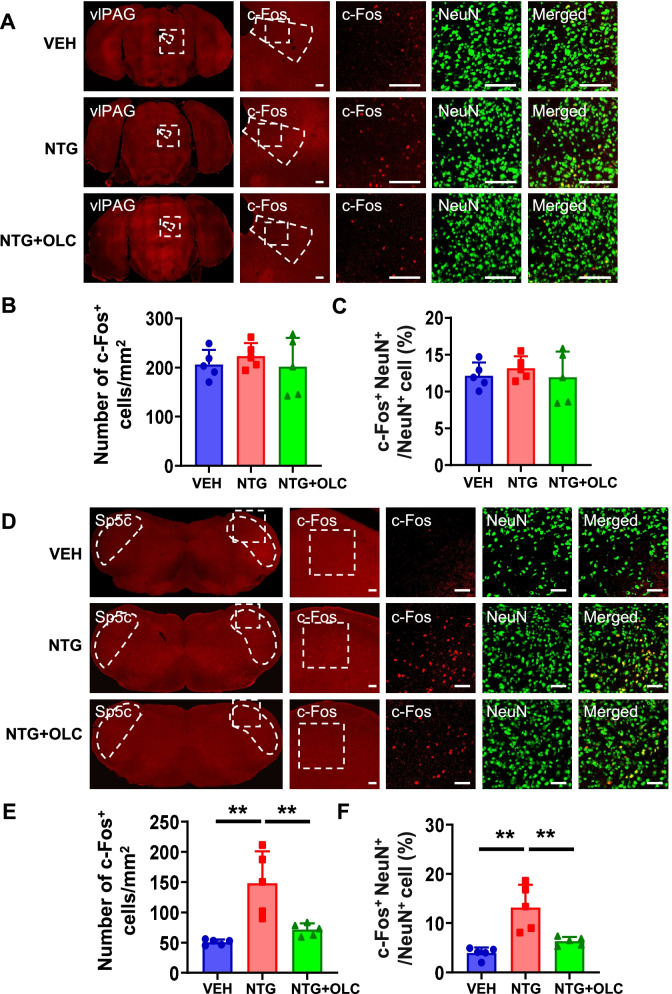
c-Fos activation map of brain stem structures in the NTG-induced chronic migraine model. **A**. Representative pictures of c-Fos (red) and NeuN (green) immunofluorescence labeling in the vlPAG. Scale bars =100 μm. **B-C**. In vlPAG, no significant differences in c-Fos^+^ cell density (**B**) and the percentage of c-Fos^+^ cells in NeuN cells (**C**) were found, alleviated by OLC, *n* = 5/group. **D.** Representative pictures of c-Fos (red) and NeuN (green) immunofluorescence labeling in the Sp5c. Scale bars =50 μm. E–F. Sp5c showed a significant increase of c-Fos^+^ cell density (**E**) and the percentage of c-Fos^+^ cells in NeuN^+^ cells (**F**). alleviated by OLC, *n* = 5/group. One-way ANOVA with the Tukey post hoc tests, * *P* < 0.05, **P < 0.01. Abbreviations: vlPAG, ventrolateral periaqueductal gray; OLC, olcegepant; Sp5c, caudal part of the spinal trigeminal nucleus

To detect the neural activity of brain regions and the role of CGRP receptors antagonist in the NTG-induced CMM, we evaluated the activation of different brain regions by using c-Fos mapping. After CMM establishment, mPFC and ACC showed a significant increase in the density of c-Fos^+^ cells and the percentage of c-Fos^+^ cells in NeuN^+^ cells, which were alleviated by OLC (*n* = 5/group, *p* < 0.01; Fig. 2A-F). In PVT and vlPAG, no significant difference was found by c-Fos mapping in the VEH, NTG, and pre-treatment with OLC group (*n* = 5/group, *p* > 0.05; Fig. 3A-C; Fig. 4 A-C). In the NTG group, Sp5c showed a significant increase in the density of c-Fos^+^ cells and the percentage of c-Fos^+^ cells in NeuN^+^ cells, which was also alleviated by OLC (*n* = 5/group, *p* < 0.01; Fig. 4 D-F).

### Olcegepant alleviated acute hyperalgesia and basal hyperalgesia in the LEV-induced chronic migraine model

**Fig. 5:**
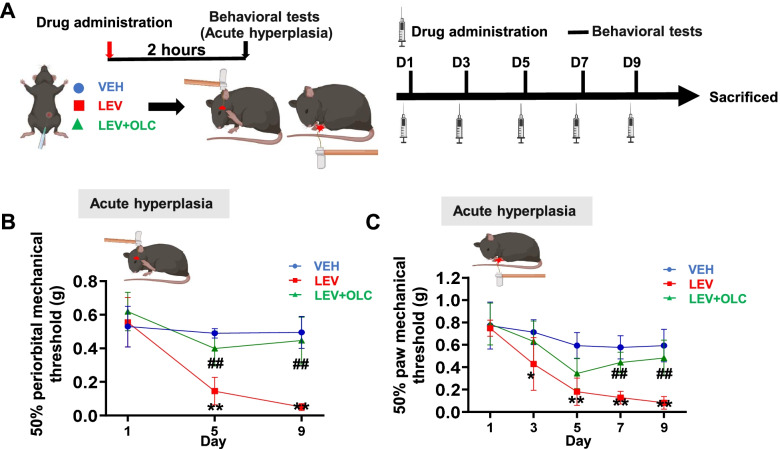
CGRP receptor antagonist (olcegepant) alleviated acute hyperalgesia in the LEV-induced chronic migraine model. **A.** Representative schematic diagrams and procedures for the behavioral tests (Created with BioRender.com). **B-C**. Repeated LEV administration induced mechanical hyperalgesia of periorbital area (**B**) and hindpaw (**C**), alleviated by OLC. Two-way ANOVA with the Tukey post hoc tests, *P < 0.05, **P < 0.01, LEV group compared with the VEH group, *n* = 8/group; #*P* < 0.05, # # *P* < 0.01, LEV group compared with the LEV+OLC group, *n* = 8/group. Abbreviations: VEH, vehicle; OLC, olcegepant; LEV, levcromakalim

Previous studies indicated that CGRP signaling as a critical factor participated in NTG and LEV-induced CMMs [22, 23]. Accordingly, mice were injected with LEV every other day for 9 days to establish CMM, and OLC was injected 15 min prior to LEV injection in other treatment groups (Fig. 5A; Fig. S1A). In the LEV-induced CMM, ANOVA analysis indicated that the mechanical thresholds of the hindpaw and periorbital area in the LEV group were significantly decreased compared with the VEH group 2 h after injection. Pre-treatment with OLC alleviated acute and basal hyperalgesia (*n* = 8/group, *p* < 0.01; Fig. 5B, C; Fig. S1B, C). These data indicated that we established a reliable LEV-induced CMM in mice and OLC alleviated acute and basal hyperalgesia.

### c-Fos activation map of cortical structures, thalamic, and brain stem structures in the LEV-induced chronic migraine model

**Fig. 6:**
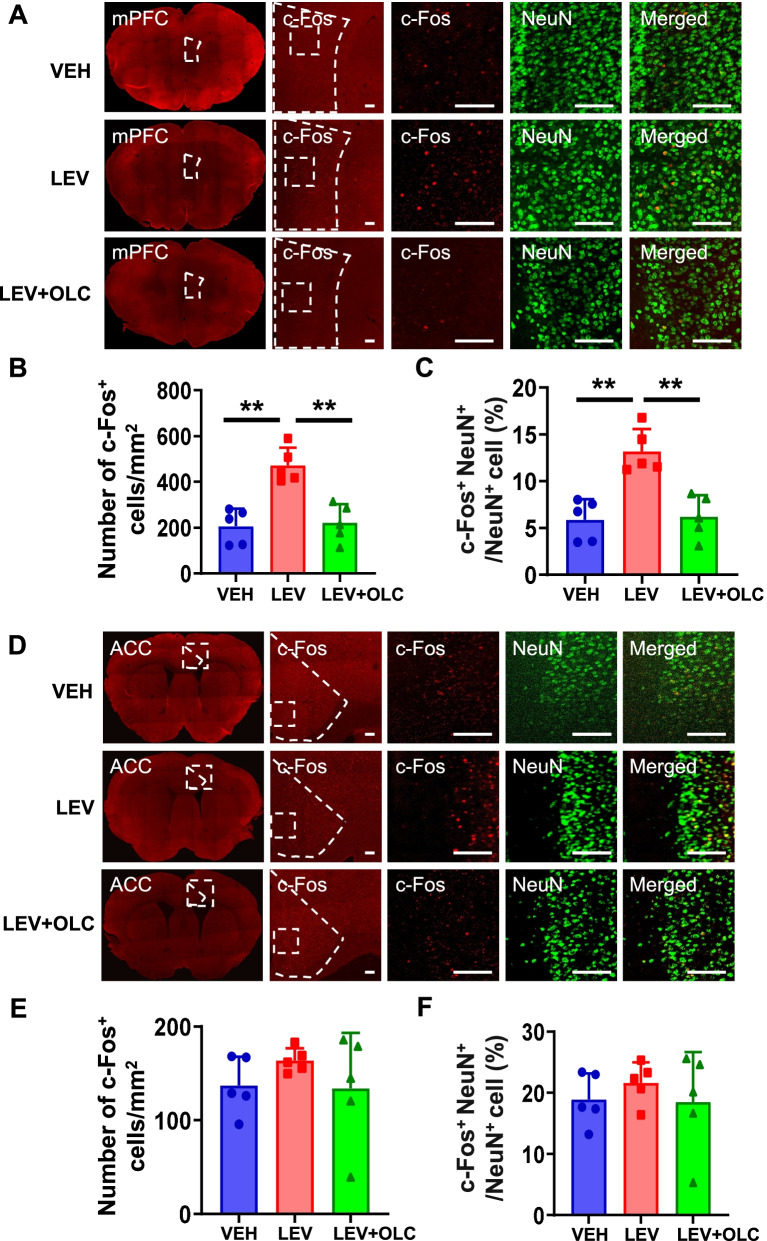
c-Fos activation map of cortical structures in the LEV-induced chronic migraine model. **A**. Representative pictures of c-Fos (red) and NeuN (green) immunofluorescence labeling in the mPFC. Scale bars =100 μm. **B-C**. mPFC showed a significant increase of c-Fos^+^ cell density (**B**) and the percentage of c-Fos^+^ cells in NeuN^+^ cells (**C**). alleviated by olcegepant. *n* = 5/group. **D**. Representative pictures of c-Fos (red) and NeuN (green) immunofluorescence labeling in the ACC. Scale bars =100 μm. **E–F.** In the ACC, no significant differences in c-Fos^+^ cell density (**E**) and the percentage of c-Fos^+^ cells in NeuN cells (**F**) were found. *n* = 5/group. One-way ANOVA with the Tukey post hoc tests, * *P* < 0.05, **P < 0.01. Abbreviations: mPFC, medial prefrontal cortex; ACC, anterior cingulate cortex; OLC, olcegepant; LEV, levcromakalim

**Fig. 7:**
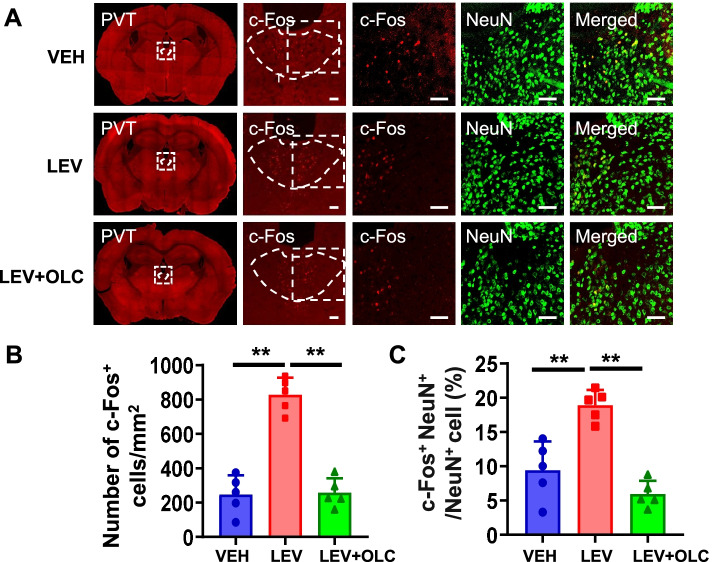
c-Fos activation map of thalamic structure in the LEV-induced chronic migraine model**. A.** Representative pictures of c-Fos (red) and NeuN (green) immunofluorescence labeling in the PVT. Scale bars =50 μm. **B-C**. PVT showed a significant increase of c-Fos^+^ cell density (**B**) and the percentage of c-Fos^+^ cells in NeuN^+^ cells (**C**), alleviated by OLC. *n* = 5/group. One-way ANOVA with the Tukey post hoc tests, * *P* < 0.05, **P < 0.01. Abbreviations: PVT, paraventricular thalamic nucleus; OLC, olcegepant. LEV, levcromakalim

**Fig. 8:**
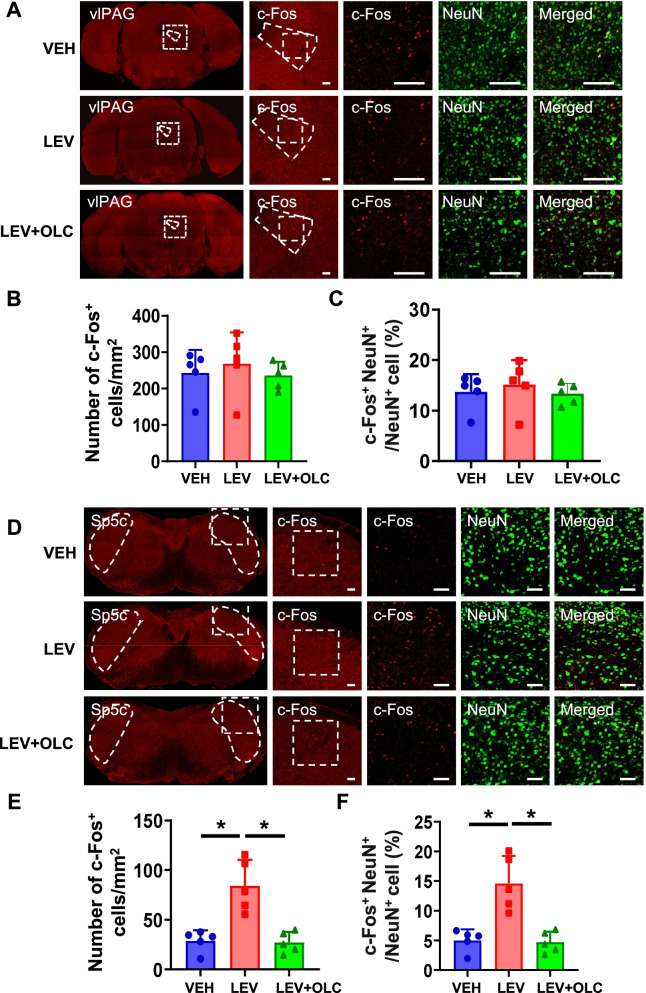
c-Fos activation map of brain stem structures in the LEV-induced chronic migraine model. **A.** Representative pictures of c-Fos (red) and NeuN (green) immunofluorescence labeling in the vlPAG. Scale bars =100 μm. **B-C.** In vlPAG, no significant differences in c-Fos^+^ cell density (**B**) and the percentage of c-Fos + cells in NeuN^+^ cells (**C**) were found. *n* = 5/group. **D.** Representative pictures of c-Fos (red) and NeuN (green) immunofluorescence labeling in the Sp5c. Scale bars =50 μm. **E–F.** Sp5c showed a significant increase of c-Fos^+^ cell density (**E**) and the percentage of c-Fos^+^ cells in NeuN^+^ cells (**F**), alleviated by OLC. *n* = 5/group. One-way ANOVA with the Tukey post hoc tests, * *P* < 0.05, **P < 0.01. Abbreviations: vlPAG, ventrolateral periaqueductal gray; OLC, olcegepant; Sp5c, caudal part of the spinal trigeminal nucleus; LEV, levcromakalim

It remains unknown whether similar activation of brain regions occurs in the LEV-induced CMM. We detected activation in the different brain regions using c-Fos and NeuN staining. After CMM establishment, mPFC showed a significant increase in the density of c-Fos^+^ cells and the percentage of c-Fos^+^ cells in NeuN^+^ cells, which were alleviated by OLC (*n* = 5/group, *p* < 0.01; Fig. 6A-C). In the ACC, no significant differences in the density of c-Fos^+^ cells and the percentage of c-Fos^+^ cells in NeuN^+^ cells were found (*n* = 5/group, *p* > 0.05; Fig. 6D-F). PVT showed a significant increase in the density of c-Fos^+^ cells and the percentage of c-Fos^+^ cells in NeuN^+^ cells after LEV treatment, which was alleviated by OLC (*n* = 5/group, *p* < 0.01; Fig. 7A-C). However, there was no significant difference in the vlPAG among the three groups (*n* = 5/group, *p* > 0.05; Fig. 8A-C). Sp5c showed a significant increase in the density of c-Fos^+^ cells and the percentage of c-Fos^+^ cells in NeuN^+^ cells in the LEV group, which was alleviated by OLC (*n* = 5/group, *p* < 0.05; Fig. 8 D-F).

## Discussion

**Fig.9:**
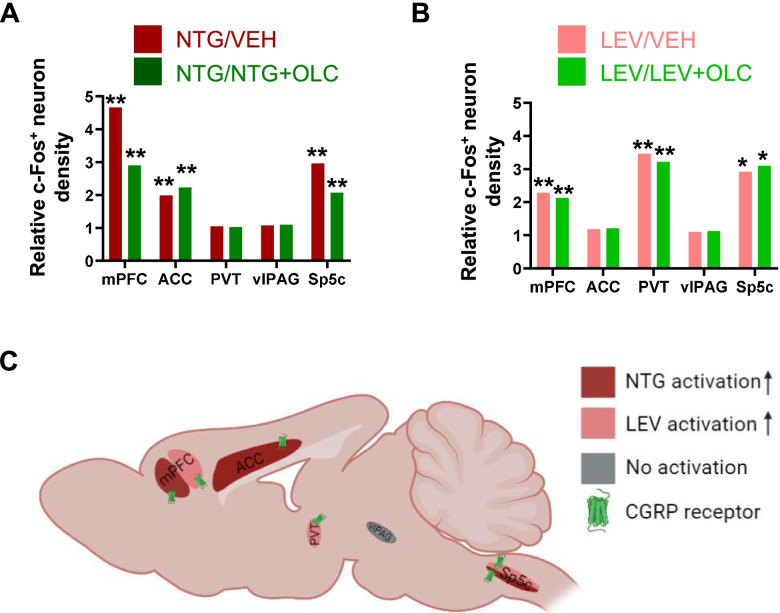
Brain activation in chronic migraine models. **A-B**. The relative c-Fos^+^ neuron density in several brain regions (A, red) and the relative c-Fos^+^ neuron density with OLC treatment (B, green) in the NTG-induced chronic migraine model or LEV-induced chronic migraine model. **C.** The brain activation and alteration of brain activation with OLC treatment in the NTG-induced chronic migraine model (dark red) and LEV-induced chronic migraine model (pink); No activation (gray); CGRP receptor (green); (Created with BioRender.com). Abbreviations: VEH, vehicle; NTG, nitroglycerin; LEV, levcromakalim; OLC, olcegepant; mPFC, medial prefrontal cortex; ACC, anterior cingulate cortex; PVT, paraventricular thalamic nucleus; vlPAG, ventrolateral periaqueductal gray; Sp5c, caudal part of the spinal trigeminal nucleus

Herein, we provide evidence that both NTG and LEV treatment can result in persistently mechanical hyperalgesia accompanied by alterations of brain activation patterns (Fig. 9 A-C). Meanwhile, OLC alleviated hyperalgesia of the hind paw and periorbital area and decreased activation in several brain regions. These alterations in brain regions help us better understanding the pathogenesis of chronic migraine and providing a potential target for the treatment of CM.

OLC alleviated acute hyperalgesia but no response in alleviating the basal hyperalgesia in the NTG-induced CMM. Recent studies have reported similar results [23, 26]. These results might be caused by a relatively short plasma half-life of OLC in rodents. However, in the LEV-induced CMM, OLC alleviated both acute and basal hyperalgesia. The activation of CGRP signaling and the opening of K_ATP_ channels were identified in both CMMs [23, 24, 33]. The different results of OLC treatment might be caused by following reasons. Firstly, NTG can activate the sensory chemoreceptor channel TRPA1 of trigeminal afferents rather than LEV [23, 34]. Secondly, NTG promotes central neuroinflammation by increasing blood–brain barrier (BBB) permeability [35]. These explained the different mechanisms contributing to migraine-like pain in two CMMs. Meanwhile, LEV can cross the BBB [36] and induce K_ATP_ channel opening, leading to the triggering of aura and migraine headache through distinct mechanisms in humans [20, 21]. It is widely thought that LEV may increase the extracellular potassium concentration in glial cells, cortical neurons, and cerebral vasculature [37] or lead to stimulation of hyperpolarization-activated cyclic nucleotide-gated cation channels [38], resulting in the generation of cortical spreading depolarization (CSD). CSD has been hypothesized to be the underlying mechanism of the migraine aura [39]. CSD might play an important role in LEV-induced CMM. OLC relieves basal hyperalgesia in the LEV-induced CMM, not in the NTG-induced CMM. A recent study showed CGRP antagonism reduces CSD, supporting the possible use of drugs targeting central CGRP receptors as antimigraine agents [40] and CGRP receptor was widely expressed in the central nervous system, including the cortex, and thalamus, PAG, and Sp5c [41–44]. Accordingly, a similar activation pattern of mPFC was found in both CMMs, owing to the shared CGRP signaling. Activation of mPFC was reversed by OLC. Meanwhile, in CM patients, resting-state functional connectivity of the default mode network also decreased in the region of interests of the lateral parietal cortex and mPFC, and headache frequency was negatively correlated with the volume of the mPFC [7, 12]. These studies also corroborated that the activation of mPFC was involved in persistent mechanical sensitization in two CMMs. In the NTG-induced CMM, the activation of ACC was consistent with clinical imaging studies and the finding in genetic migraine models. For instance, in CM patients, stronger structural connectivity was found between the caudal ACC and other brain regions [5, 10] and the N-acetyl-aspartate of bilateral thalami and right ACC decreased [6]. In the familial hemiplegic migraine type 2 mouse model, migraine-relevant hypersensitivity triggered by NTG has been attributed to the alteration of neural function in the cingulate cortex [45]. However, unlike NTG, in the LEV-induced CMM, no significant difference of c-Fos expression in ACC was found. Moreover, in the NTG-induced CMM with early growth response gene 1 (Egr1)-enhanced green fluorescent protein transgenic mice, no significant difference in Egr1 expression was found in the ACC [18]. These results were also explained by the analysis of different IEG markers of active neurons.

Thalamic central sensitization maybe contributes to the chronification of migraine [4]. In this study, neuronal activity of the PVT increased in the LEV-induced CMM. It is well-known that PVT plays a critical role in the central processing of chronic pain [46]. However, no alteration in neuronal activity of PVT was found in the NTG-induced CMM. The PVT-CeA-vlPAG circuit reportedly mediates the central mechanisms of descending pain facilitation underlying persistent pain [46]. Although several clinical studies showed that vlPAG, the descending pain-modulating system, was associated with allodynia [15, 47], vlPAG did not exhibit a significant difference between two CMMs. In other rat migraine models, CSD decreased c-Fos expression in PAG [48], and inflammatory soup administration increased c-Fos expression in PAG [49]. Thus, the mechanism of vlPAG and PVT contributing to migraine-like pain was different in CMMs. Interestingly, neurons in Sp5c were activated in two CMMs. CM patients also exhibited cephalic and extracephalic allodynia, corresponding to the sensitization of the second-order neurons in the Sp5c [50]. Recent studies have also reported microglia activation and aberrant synaptic plasticity in the Sp5c contribute to central sensitization in the NTG-induced CMM [29, 51, 52]. Our results further substantiated that the activation of Sp5c may be involved in persistent mechanical sensitization. As expected, OLC decreased the activation of Sp5c. Immunohistochemical studies performed with rat and human tissue revealed that the CGRP receptor was expressed in the medulla-pons region, and cervical spinal cord [41, 44]. NTG increased the gene expression of CGRP and c-Fos in the Sp5c. Our findings suggest that OLC may act on the CGRP receptor of the Sp5c to attenuate neuronal activity.

There were several strengths and limitations in this study. To the best of our knowledge, this is the first study to report the c-Fos activation map of brain regions in NTG and LEV-induced CMMs and provided a reference for neural circuit study in future research. However, we only focused on five representative coronal sections. These results not necessarily were observed in CM patients, and the function of other brain regions in CMM can’t be excluded entirely. Besides, a recent study showed that basal hyperalgesia was maintained for a week after NTG injection on day 9 [30]. We only focused on the c-Fos of expression in brain regions after NTG injection on day 9, but whether these brain regions are continuously activated after the last NTG/LEV injection need further to be explored.

## Conclusions

In conclusion, our study demonstrated the activation of mPFC and Sp5c in both CMMs. OLC may alleviate hyperalgesia of the hind paw and periorbital area by attenuating brain activation in CMMs.

## Supplementary Information

**Additional file 1: Figure S1.** CGRP receptor antagonist (olcegepant) did not alleviate basal hyperalgesia in the NTG-induced chronic migraine model. **A. **Representative schematic diagrams and procedures for the behavioral tests. **B-C.** Repeated NTG administration induced basal hyperalgesia of periorbital area (**B**) and hindpaw (**C**), but not alleviated by OLC. Two-way ANOVA with the Tukeypost hoc tests; * P<0.05, **P<0.01, NTG group compared with the VEH group, n=8/group; #P<0.05, ## P<0.01, NTG group compared with the NTG+OLC group, n=8/group. Abbreviations: VEH, vehicle; NTG, nitroglycerin; OLC, olcegepant. **Figure S2.** CGRP receptor antagonist (olcegepant) alleviated basal hyperalgesia in the LEV-induced chronic migraine model. **A.** Representative schematic diagrams and procedures for the behavioral tests. **B-C. **Repeated LEV administration induced mechanical hyperalgesia of periorbital area (**B**) and hindpaw (**C**) alleviated by OLC. Two-way ANOVA with the Tukey post hoc tests; * P<0.05, **P<0.01, LEV group compared with the VEH group, n=8/group; #P<0.05, ## P<0.01, LEV group compared with the LEV+OLC group, n=8/group. Abbreviations: VEH, vehicle; NTG, nitroglycerin; OLC, olcegepant; LEV, levcromakalim.

## Data Availability

The data used and analyzed in this article are available upon reasonable request.

## Abbreviations

ACC: Anterior cingulate cortex
ANOVA: Analysis of variance
BBB: Blood–brain barrier
CM: Chronic migraine
CMM: Chronic migraine model
CSD: Cortical spreading depolarization
CGRP: Calcitonin gene-related peptide
DMSO: Dimethyl sulfoxide
Egr1: Early growth response gene 1
IEG: Immediate-early gene
K_ATP_: ATP-sensitive potassium channel opener
LEV: Levcromakalim
mPFC: Medial prefrontal cortex
NeuN: Neuronal nuclei
NTG: Nitroglycerin
OLC: Olcegepant
PVT: Paraventricular thalamic nucleus
Sp5c: Caudal part of the spinal trigeminal nucleus
SEM: Standard error of the mean
vlPAG: Ventrolateral periaqueductal gray
VEH: Vehicle
