# Supplementary figures and images for: A c-Fos activation map in nitroglycerin/levcromakalim-induced models of migraine
Source: J Headache Pain. 2022 Sep 30;23(1):128. doi: 10.1186/s10194-022-01496-8 (PMC9524028; doi:10.1186/s10194-022-01496-8)

# FigS1

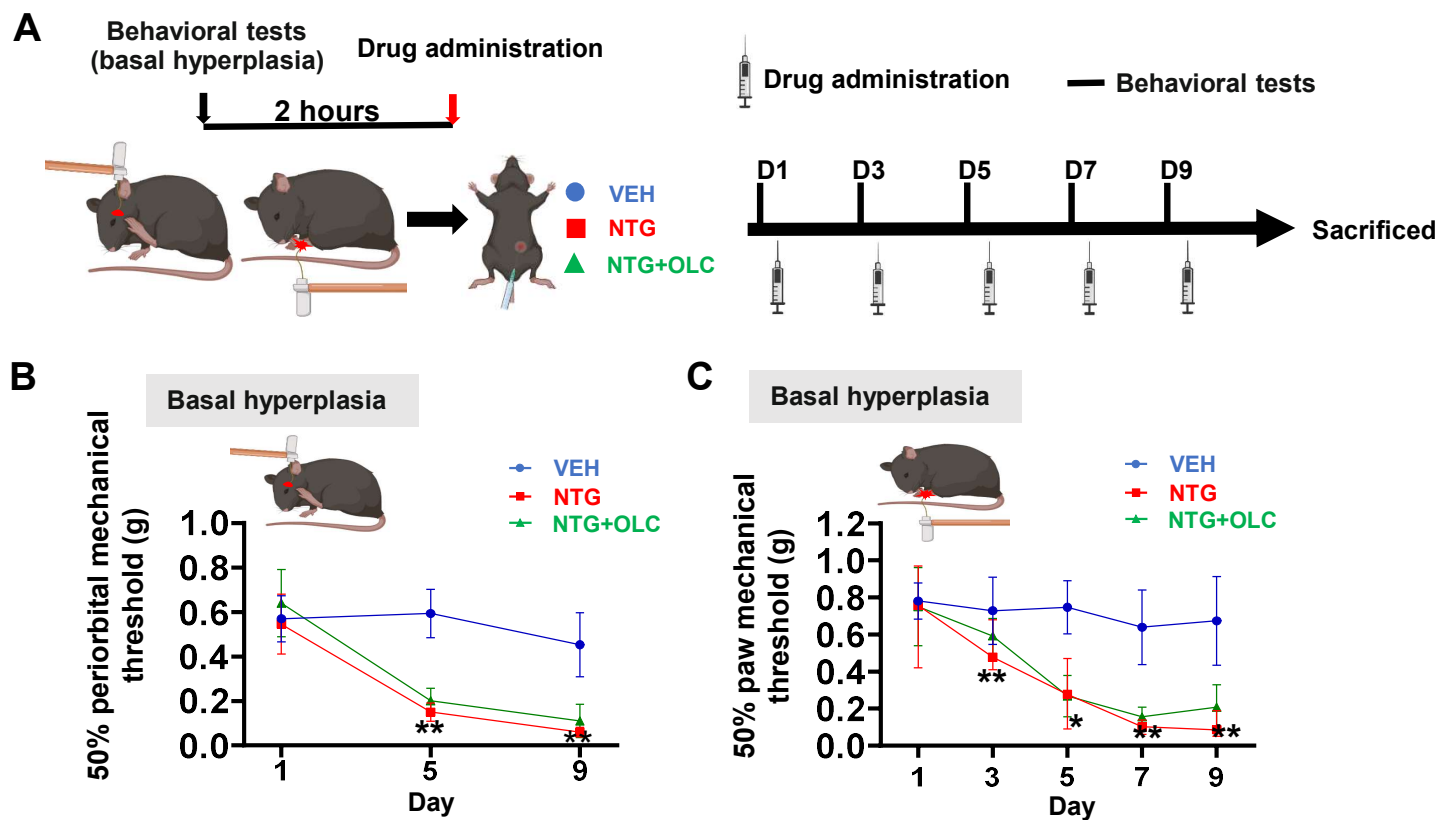

FigS2

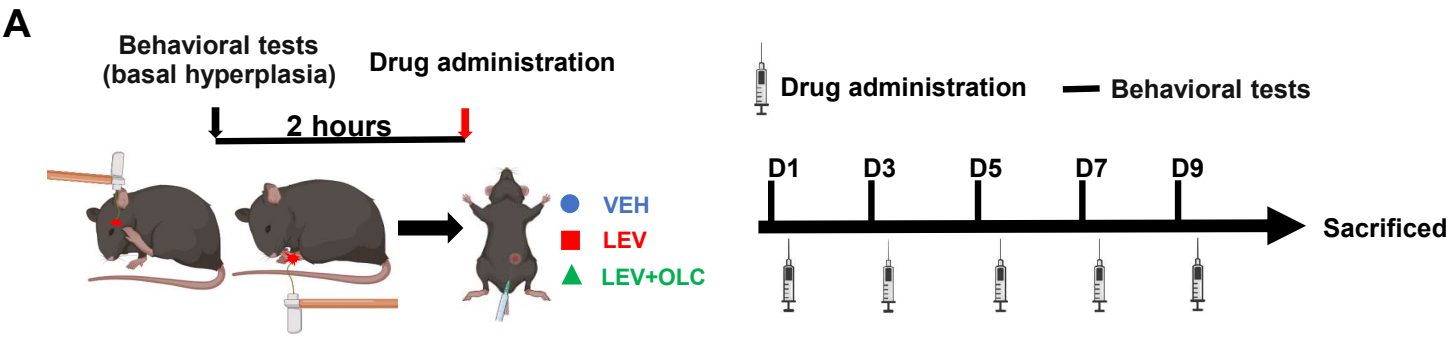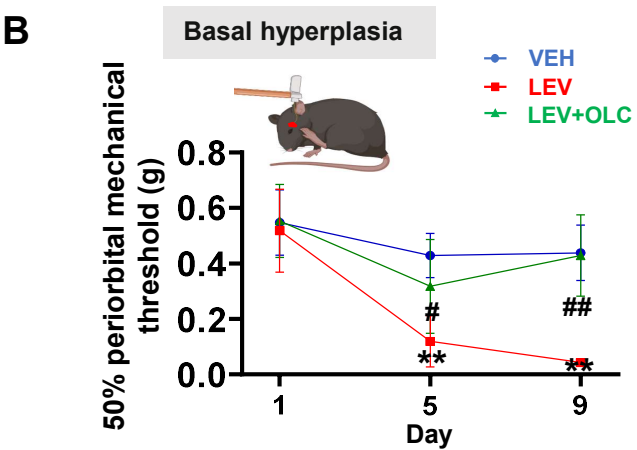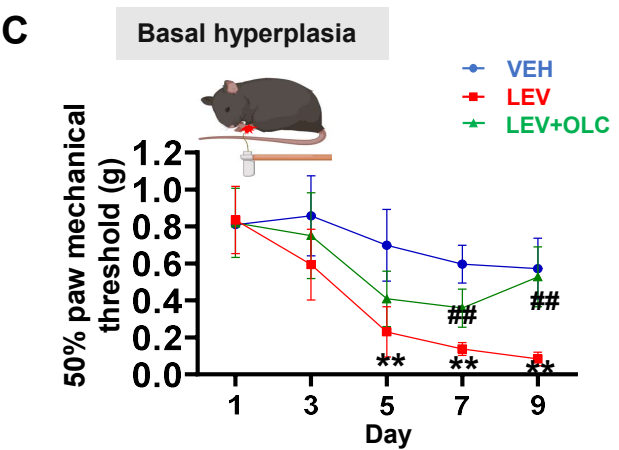

Supplement: Supplementary file 1 — Additional file 1: Figure S1. CGRP receptor antagonist (olcegepant) did not alleviate basal hyperalgesia in the NTG-induced chronic migraine model. A. Representative schematic diagrams and procedures for the behavioral tests. B-C. Repeated NTG administration induced basal hyperalgesia of periorbital area (B) and hindpaw (C), but not alleviated by OLC. Two-way ANOVA with the Tukeypost hoc tests; * P<0.05, **P<0.01, NTG group compared with the VEH group, n=8/group; #P<0.05, ## P<0.01, NTG group compared with the NTG+OLC group, n=8/group. Abbreviations: VEH, vehicle; NTG, nitroglycerin; OLC, olcegepant. Figure S2. CGRP receptor antagonist (olcegepant) alleviated basal hyperalgesia in the LEV-induced chronic migraine model. A. Representative schematic diagrams and procedures for the behavioral tests. B-C. Repeated LEV administration induced mechanical hyperalgesia of periorbital area (B) and hindpaw (C) alleviated by OLC. Two-way ANOVA with the Tukey post hoc tests; * P<0.05, **P<0.01, LEV group compared with the VEH group, n=8/group; #P<0.05, ## P<0.01, LEV group compared with the LEV+OLC group, n=8/group. Abbreviations: VEH, vehicle; NTG, nitroglycerin; OLC, olcegepant; LEV, levcromakalim. [file 10194_2022_1496_MOESM1_ESM.pdf]
